# Edible coatings and films in meat preservation: Materials, functional mechanisms, and technological challenges

**DOI:** 10.14202/vetworld.2026.3311-3336

**Published:** 2026-08-04

**Authors:** Bima Putra Pratama, Aswin Rafif Khairullah, Mustofa Helmi Effendi, Budiastuti Budiastuti, Dian Ayu Permatasari, Wiwiek Tyasningsih, Saifur Rehman, Emmanuel Nnabuike Ugbo, Ikechukwu Benjamin Moses, Dea Anita Ariani Kurniasih, Bantari Wisynu Kusuma Wardhani, Ilma Fauziah Ma'ruf, Sri Suryatmiati Prihandani, Erfina Febrianti

**Affiliations:** 1 Research Center for Process Technology, National Research and Innovation Agency (BRIN), KST BJ Habibie, Serpong, South Tangerang, Banten, 15314, Indonesia.; 2 Research Center for Veterinary Science, National Research and Innovation Agency (BRIN), Jl. Raya Bogor Km. 46 Cibinong, Bogor, West Java, 16911, Indonesia.; 3 Division of Veterinary Public Health, Faculty of Veterinary Medicine, Universitas Airlangga, Kampus C Mulyorejo, Jl. Dr. Ir. H. Soekarno, Surabaya, East Java, 60115, Indonesia.; 4 Study Program of Pharmacy Science, Faculty of Health Science, Universitas Muhammadiyah Surabaya, Jl. Raya Sutorejo No.59, Dukuh Sutorejo, Mulyorejo, Surabaya, East Java, 60113, Indonesia.; 5 Division of Veterinary Microbiology, Faculty of Veterinary Medicine, Universitas Airlangga, Kampus C Mulyorejo, Jl. Dr. Ir. H. Soekarno, Surabaya, East Java, 60115, Indonesia.; 6 Department of Pathobiology, Faculty of Veterinary and Animal Sciences, Gomal University, RV9W+GVJ, Indus HWY, Dera Ismail Khan, 27000, Pakistan.; 7 Department of Applied Microbiology, Faculty of Science, Ebonyi State University, Abakaliki Rd, Abakaliki, Ebonyi, 481101, Nigeria.; 8 Research Center for Public Health and Nutrition, National Research and Innovation Agency (BRIN), Jl. Raya Bogor Km. 46 Cibinong, Bogor, West Java, 16911, Indonesia.; 9 Research Center for Pharmaceutical Ingredients and Traditional Medicine, National Research and Innovation Agency (BRIN), Jl. Raya Bogor Km. 46 Cibinong, Bogor, West Java, 16911, Indonesia.; 10 Department of Agroindustrial Technology, Faculty of Industrial Technology, Institut Teknologi Sumatera, Jl. Terusan Ryacudu, Way Huwi, Jati Agung, Lampung, 35365, Indonesia.

**Keywords:** active packaging, biopolymer, edible coating, edible film, meat preservation, nanoencapsulation, shelf-life extension, smart packaging

## INTRODUCTION

Meat is a major source of high-quality animal protein and essential nutrients; however, it is highly susceptible to deterioration during processing, distribution, and storage because of physical, chemical, and microbiological factors [1]. Maintaining meat quality and safety is essential for protecting consumer health, sustaining the economic value of the meat industry, and supporting global food security [2]. Meat deterioration is primarily associated with lipid oxidation, myoglobin pigment degradation, microbial proliferation, and the subsequent loss of desirable sensory attributes, including color, texture, and flavor [3]. Lipid oxidation generates peroxides, aldehydes, and ketones that contribute to off-flavors, nutritional losses, and reduced shelf-life, whereas myoglobin oxidation diminishes the visual appeal of meat products [4]. Furthermore, contamination and proliferation of foodborne pathogens, including *Listeria monocytogenes*, *Salmonella* spp., and *Escherichia coli*, pose significant food safety concerns, particularly in fresh and ready-to-eat meat products [5].

Conventional preservation strategies, including refrigeration, freezing, vacuum packaging, and synthetic preservatives, are widely used to extend the shelf-life of meat products. Nevertheless, these approaches have important limitations, including concerns regarding environmental sustainability, chemical residues, packaging waste, and potential adverse effects on sensory quality [6]. Growing consumer preference for natural, safe, and environmentally sustainable foods has therefore accelerated research into edible coatings and films as promising alternatives to conventional preservation technologies [7]. These edible systems form thin protective layers or standalone wraps around meat products, acting as semipermeable barriers that reduce O_2_ and water vapor transmission, retard lipid oxidation, minimize moisture loss, and suppress microbial growth [8]. Moreover, edible coatings and films can serve as carriers for bioactive compounds, including antimicrobial agents, antioxidants, and probiotics, thereby functioning as active packaging systems with multiple preservation mechanisms [9].

Edible coatings and films are commonly formulated from protein-, polysaccharide-, and lipid-based biopolymers. Protein-based materials, such as gelatin, whey protein, and soy protein, generally provide excellent mechanical strength and gas barrier properties, whereas polysaccharides, including chitosan, alginate, and pectin, offer superior O_2_ barrier characteristics and film-forming ability. Lipid-based materials primarily contribute hydrophobic properties that reduce water vapor permeability [10, 11]. Composite formulations combining proteins, polysaccharides, and lipids can overcome the limitations of individual biopolymers, resulting in improved barrier performance, flexibility, and mechanical stability [12]. Furthermore, incorporation of functional bioactive compounds through technologies such as nanoencapsulation enhances the stability, controlled release, bioavailability, antimicrobial efficacy, and antioxidant performance of edible coating systems [13].

Recent technological advances have transformed edible coatings and films from passive barrier materials into multifunctional active and intelligent packaging systems capable of preserving meat quality while simultaneously monitoring freshness and product quality [14]. These innovations not only improve food safety and shelf-life extension but also support sustainable food production by reducing reliance on synthetic preservatives and conventional petroleum-based packaging materials, thereby meeting increasing consumer demand for environmentally responsible preservation technologies [15].

Despite considerable progress in the development of edible coatings and films, several important knowledge gaps remain. Existing reviews have predominantly focused on individual coating materials, specific bioactive compounds, or isolated preservation mechanisms, with relatively limited integration of recent advances in composite and nanocomposite formulations, smart edible packaging, nanoencapsulation technologies, and their practical application across different meat systems. Furthermore, the growing body of evidence regarding multifunctional coatings, integration with emerging preservation technologies such as modified atmosphere packaging, high-pressure processing, ultraviolet treatment, and cold plasma, as well as challenges related to industrial scalability, regulatory compliance, consumer acceptance, and commercial implementation, has not been comprehensively synthesized. Consequently, a contemporary review that critically integrates material selection, functional mechanisms, technological innovations, practical applications, evaluation methods, and future development priorities is needed to provide a comprehensive understanding of the current state of edible coating technologies for meat preservation.

This review aims to provide a comprehensive and up-to-date synthesis of edible coatings and films for meat preservation. Specifically, it discusses the classification and characteristics of edible coating materials, their functional mechanisms, applications in fresh and processed meat products, methods used to evaluate preservation efficacy, and the major technological challenges limiting industrial implementation. In addition, the review highlights recent advances in multifunctional edible coatings, nanotechnology, smart packaging systems, and integration with complementary preservation technologies while identifying current research gaps and future perspectives. By critically consolidating the available evidence, this review seeks to provide researchers, food scientists, and the meat industry with a scientific foundation for developing sustainable, safe, and effective edible coating technologies that enhance meat quality, extend shelf-life, and support next-generation active food packaging systems.

## LITERATURE SEARCH AND DATA COLLECTION

This review was conducted as a structured narrative (scoping-oriented) literature synthesis to provide a comprehensive overview of edible coatings and edible films for meat preservation, encompassing biomaterial classifications, mechanisms of action, applications in fresh and processed meat products, evaluation methodologies, and current technological challenges. A systematic literature search was performed using the major scientific databases Scopus, Web of Science, PubMed, and Google Scholar, covering publications from 2015 to 2025 to capture both foundational studies and the most recent advances in the field. The search strategy employed combinations of the following keywords: edible coating*, *edible film*, *meat*, *poultry*, *processed meat*, *active packaging*, *antimicrobial*, *antioxidant*, *essential oils*, *biopolymers*, *protein*, *polysaccharide*, *lipid*, *composite*, *nanocomposite*, *nanoencapsulation*, *modified atmosphere packaging (MAP)*, *high-pressure processing (HPP)*, *and shelf-life*.*

Priority was given to peer-reviewed articles published in English to ensure inclusion of high-quality and contemporary evidence, whereas seminal earlier publications were retained when necessary to support fundamental concepts and definitions. Titles and abstracts were initially screened for relevance to edible coatings and edible films applied to meat systems, followed by full-text evaluation to confirm eligibility. Studies focusing primarily on non-meat food matrixes, lacking preservation-related outcome measures, containing duplicate datasets, or providing insufficient methodological information were excluded.

**Relevant information from eligible studies was extracted and organized according to:** (i) coating formulation, biopolymer type, and functional additives; (ii) target meat products and storage or processing conditions; and (iii) preservation outcomes, including chemical indicators (e.g., thiobarbituric acid reactive substances [TBARS], pH, and water-holding capacity (WHC)), microbiological parameters (e.g., total plate count (TPC) and specific foodborne pathogens), sensory characteristics, and shelf-life evaluation. The extracted evidence was synthesized comparatively to identify emerging trends, technological advantages, existing limitations, research gaps, and practical considerations related to regulatory compliance, food safety, consumer acceptance, and industrial scalability.

## CONCEPT AND CLASSIFICATION OF EDIBLE COATINGS AND FILMS

Edible coatings and edible films are biopolymer based preservation technologies designed for direct application to food products, providing an additional protective layer that is safe for consumption together with the food [16, 17]. Both systems function as protective barriers that regulate mass transfer, including the diffusion of gases, water vapor, and volatile compounds, while also providing protection against mechanical damage and microbial contamination [18]. Their application in meat preservation has become increasingly important because meat is highly susceptible to lipid oxidation, discoloration, moisture loss, and microbial proliferation, all of which contribute to quality deterioration and reduced shelf-life [19].

Table 1 summarizes the principal biopolymer groups used in edible coatings and edible films, highlighting their physicochemical properties, technological functions, and preservation mechanisms in meat products [16–19].

Although edible coatings and edible films perform similar preservation functions, they differ considerably in their preparation methods and physical characteristics. Edible coatings are applied directly onto food surfaces by dipping, spraying, or brushing, thereby forming a thin, adherent protective layer [20]. In contrast, edible films are manufactured separately as free-standing sheets through casting or extrusion before being applied as wraps or incorporated into active packaging systems [21]. These differences in fabrication methods influence film thickness, mechanical strength, flexibility, and diffusion barrier properties, ultimately determining their suitability for different meat products and storage conditions [22].

**Table 1:** Characteristics, technological functions, and protective mechanisms of major biopolymers used in edible coatings and films for meat preservation [16–19].

**Biopolymer group**	**Examples**	**Physicochemical and mechanical properties**	**Technological function**	**Protective mechanisms in meat preservation**
Protein-based	Gelatin, whey protein, soy protein	Flexible, high tensile strength, good adhesion, excellent film-forming ability, and dense polymeric matrix	Forms edible coatings by dipping, spraying, or brushing, and edible films by casting or extrusion that adhere uniformly to meat surfaces	Acts as an effective oxygen barrier, delays lipid oxidation, preserves myoglobin stability and meat color, and serves as a carrier matrix for antioxidants and antimicrobial compounds
Polysaccharide-based	Chitosan, alginate, starch, cellulose derivatives	Low oxygen permeability, strong gel-forming capacity, high transparency, good structural integrity, and moderate flexibility	Produces stable edible coatings and films suitable for refrigerated storage while regulating gas and moisture transfer	Suppresses microbial growth (particularly through the antimicrobial activity of chitosan), reduces moisture loss, delays spoilage, and extends shelf-life
Lipid-based	Beeswax, fatty acids, glycerol lipids	Hydrophobic, excellent water vapor barrier properties, relatively low flexibility, and semi-transparent appearance	Functions primarily as a moisture barrier, either alone or as a component of composite edible coatings and films	Minimizes moisture loss, maintains meat juiciness, reduces dehydration, and limits oxidative deterioration during storage

Composite films = Edible films produced by combining two or more classes of biopolymers (e.g., proteins, polysaccharides, and lipids) to improve barrier, mechanical, and functional properties.

**Figure 1:**
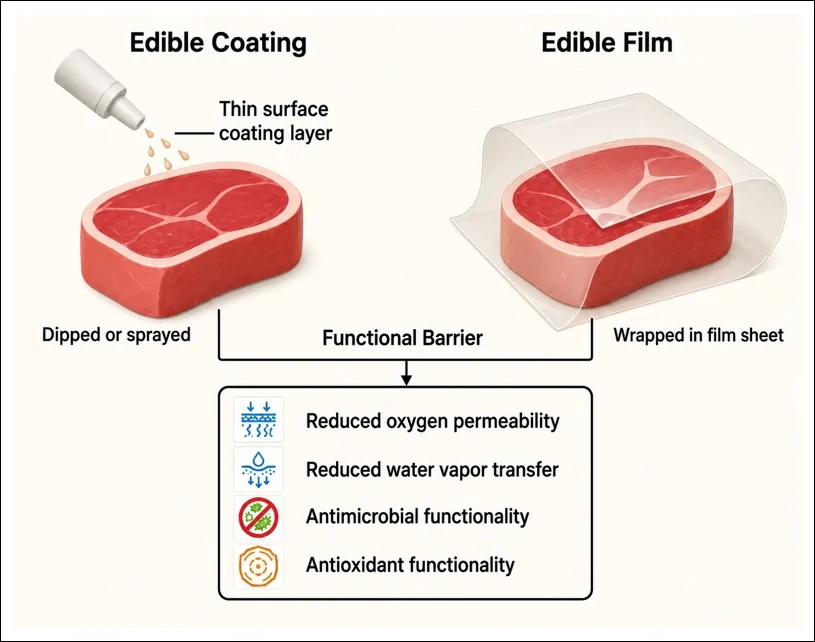
Conceptual overview of edible coatings and edible films used for meat preservation. [Source*:* Figure prepared by Bima Putra Pratama, with the assistance of ChatGPT (OpenAI, GPT-5.5) for AI-assisted figure generation and Canva for graphic design and final layout].

Figure 1illustrates the fundamental differences between edible coatings, which are formed directly on the meat surface by dipping, spraying, or brushing, and edible films, which are preformed separately and subsequently applied to meat products. Both systems function as protective barriers that reduce O_2_ and moisture transfer, retard lipid oxidation, suppress microbial growth, preserve sensory attributes, and extend the shelf-life of fresh and processed meat products.

## REGULATORY DEFINITIONS AND APPLICATION METHODS

Edible coatings and edible films are classified as food-contact materials by regulatory authorities, including the U.S. Food and Drug Administration (FDA) and the European Food Safety Authority (EFSA) [23]. The FDA recognizes edible coating materials as food additives provided they comply with established safety requirements. For example, biopolymers such as gelatin, chitosan, and alginate are generally recognized as safe for application to meat products [24]. Similarly, the EFSA evaluates the safety of edible coating materials under Regulation (EC) No. 1935/2004, which requires that food-contact materials do not pose risks to consumer health.

Edible coatings and edible films are further classified according to their application techniques, including dipping, spraying, brushing, casting, and extrusion, as well as their physical thickness [24]. Edible coatings typically form thin, uniform layers measuring approximately 5–100 μm, whereas edible films are generally thicker (0.1–1.0 mm) because they are produced as independent sheets for wrapping or incorporation into packaging systems [25]. The selected application method and film thickness directly influence the mechanical strength, barrier performance, flexibility, and overall preservation efficacy of the edible material [26].

## MATERIALS USED FOR EDIBLE COATINGS AND FILMS

**Figure 2:**
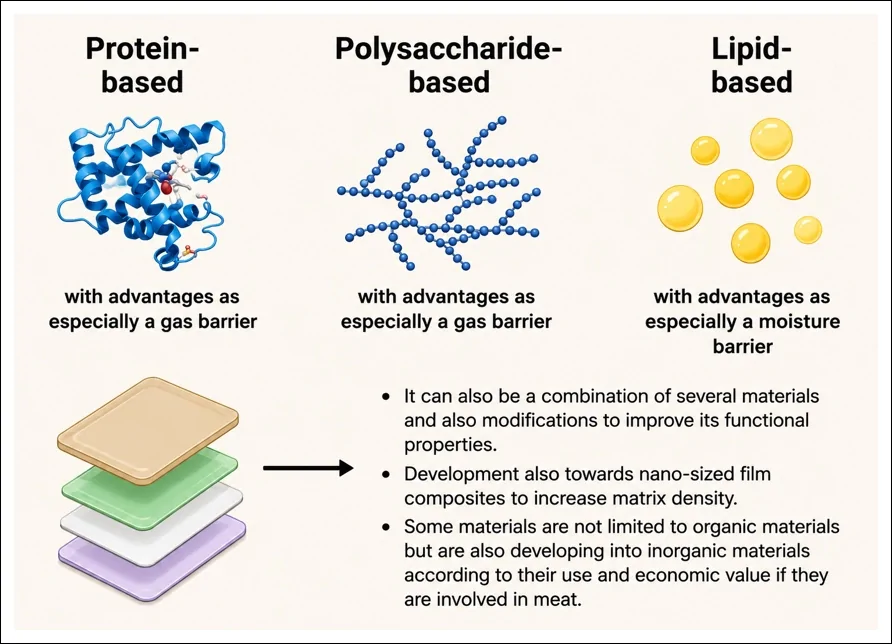
Major classes of biopolymers used in edible coatings and edible films for meat preservation. [Source: Figure prepared by Bima Putra Pratama, with the assistance of ChatGPT (OpenAI, GPT-5.5) for AI-assisted figure generation and Canva for graphic design and final layout].

The effectiveness of edible coatings and edible films in meat preservation is largely determined by the selection of appropriate constituent materials [27]. These materials not only serve as the structural matrix but also influence the mechanical properties, barrier performance, and functional characteristics of the coating, including antimicrobial and antioxidant activities [28]. The use of individual biopolymers, composite formulations, and nanocomposite technologies enriched with functional additives has enabled the development of multifunctional coating systems capable of extending shelf-life while maintaining the physicochemical, microbiological, and sensory quality of meat products [29]. Figure 2categorizes the principal biopolymer groups, including protein-based, polysaccharide-based, and lipid-based materials, and highlights representative examples together with their key physicochemical characteristics and functional roles in enhancing barrier properties, mechanical stability, and preservation efficacy.

### Biopolymers

Biopolymers constitute the primary structural components of edible coatings and edible films, forming a protective matrix on the surface of meat products [30]. These materials are generally classified into three major groups: protein-based, polysaccharide-based, and lipid-based biopolymers, each possessing distinct physico-chemical properties, technological functions, and preservation mechanisms that contribute to reducing meat deterioration [31]. Selection of an appropriate biopolymer depends on the intended application, storage conditions, and desired quality attributes of the final meat product [32].

Protein-based biopolymers, including gelatin, whey protein, and soy protein, produce films with excellent mechanical properties, such as high tensile strength, flexibility, and strong adhesion to meat surfaces [33]. Protein molecules form cohesive three-dimensional networks through hydrogen bonding and covalent interactions, thereby reducing O_2_ permeability and delaying lipid oxidation [34]. In addition, protein-based coatings effectively encapsulate and deliver bioactive compounds, including antioxidants and antimicrobial agents, thereby enhancing their preservation efficiency [35].

Polysaccharide-based biopolymers, such as chitosan, alginate, starch, and cellulose derivatives, exhibit excellent film-forming ability, low O_2_ permeability, high transparency, and favorable gel-forming characteristics [36]. Chitosan possesses inherent antimicrobial activity through electrostatic interactions with negatively charged bacterial cell membranes, leading to disruption of membrane integrity and inhibition of microbial growth [37]. Furthermore, polysaccharide-based coatings remain stable under refrigerated storage conditions, making them particularly suitable for fresh and processed meat products [38].

Lipid-based biopolymers, including beeswax, fatty acids, and lipid derivatives, primarily function as hydrophobic barriers that effectively reduce water vapor transmission and moisture loss [39]. Their hydrophobic nature makes them valuable components of multilayer edible coating systems designed to preserve juiciness and minimize dehydration during storage [40]. However, lipid-based films generally exhibit lower mechanical strength and transparency than protein- and polysaccharide-based films; consequently, they are more commonly incorporated as external layers within composite formulations [41].

Combining these three classes of biopolymers through composite and nanostructured formulations produces coatings with more balanced mechanical properties and improved barrier performance that are better suited to the complex preservation requirements of meat products [42]. Nevertheless, large-scale industrial implementation requires consideration of production costs, raw material availability, manufacturing scalability, and economic feasibility. The production cost of protein- and polysaccharide-based coatings may be reduced by utilizing agricultural and food-processing by-products as renewable raw materials. In addition, the potential allergenicity associated with ingredients such as whey protein and soy protein necessitates appropriate labeling to ensure consumer safety [43].

Biodegradability is another important characteristic of biopolymer based coatings that supports environ-mental sustainability. Although these materials provide environmentally friendly alternatives to conventional synthetic packaging, further investigation into degradation kinetics, recycling potential, and industrial processing is needed to facilitate their commercial application. Overall, biopolymer based edible coatings and films represent sustainable preservation systems capable of extending shelf-life, improving food safety, and maintaining the sensory quality of meat products.

### Composite and nanocomposite films

Recent advances in edible coating technology have increasingly focused on composite and nanocomposite formulations to improve mechanical properties, gas barrier performance, and structural stability [44]. Composite systems combine two or more polymers, such as protein-polysaccharide, polysaccharide-lipid, or protein-lipid formulations, to compensate for the limitations of individual materials [45]. For example, combining proteins with polysaccharides enhances tensile strength while reducing O_2_ permeability, whereas incorporation of lipid components effectively decreases water vapor transmission [46]. These synergistic interactions produce films with balanced barrier characteristics that better preserve the physicochemical and sensory quality of meat during storage [47].

Nanocomposite technology further enhances edible film performance through incorporation of nano-structured materials, including nanochitosan, nanoclay, lipid nanoemulsions, and naturally derived nanoparticles [48]. These nanomaterials strengthen the polymer matrix while improving antimicrobial activity, antioxidant stability, mechanical properties, and thermal resistance [49]. Nanochitosan exhibits enhanced antimicrobial activity because of its greater ability to interact with and penetrate microbial cell membranes, whereas plant-derived nanoparticles containing bioactive compounds effectively reduce lipid oxidation and protein degradation during refrigerated storage [50]. Similarly, essential oil nanoemulsions improve the solubility, stability, and controlled release of antimicrobial and antioxidant compounds within the polymer matrix, thereby providing simultaneous protection against oxidative deterioration and microbial spoilage [51, 52]. The incorporation of nanoclay also enhances O_2_ and water vapor barrier properties by increasing the tortuosity of diffusion pathways within the polymer network [53].

**Safety and regulatory considerations of nanocomposites:** Despite their technological advantages, nano-composite edible films present several safety and regulatory challenges that require careful consideration. One major concern is the potential migration of nanoparticles from packaging materials into meat products, which may present toxicological risks if migration exceeds acceptable safety limits. Although currently available evidence indicates that certain nanomaterials, such as nanochitosan, exhibit favorable safety profiles, comprehensive long-term toxicological evaluations remain limited [54]. Consequently, rigorous regulatory assessment is essential to ensure that nanomaterials used in food applications comply with established safety standards developed by regulatory authorities, including the FDA and EFSA [55].

Consumer acceptance also represents an important challenge for commercialization. Public concerns regarding the safety of nano-enabled food packaging, together with the absence of harmonized international regulations governing nanomaterials in foods, continue to limit widespread adoption. Nevertheless, when supported by comprehensive safety assessments, transparent regulatory frameworks, and effective public education, nanocomposite technologies offer substantial benefits for extending shelf-life, reducing food spoilage, and improving food safety.

### Functional additives

Functional additives play an essential role in enhancing the performance of edible coatings and edible films by transforming passive barrier systems into active preservation technologies capable of minimizing oxidative deterioration and microbial contamination [54]. Common functional additives include antimicrobial agents, antioxidants, plasticizers, and emulsifiers, all of which improve the physicochemical stability and functional performance of edible films during storage [55]. The selection of these additives depends on the characteristics of the biopolymer matrix, storage conditions, and the intended quality attributes of the final meat product [56].

Antimicrobial additives commonly incorporated into edible coatings include essential oils, plant extracts, and probiotics [57]. Essential oils derived from thyme, oregano, and rosemary contain bioactive constituents such as carvacrol and thymol that disrupt bacterial cell membranes, impair cytoplasmic integrity, and inhibit essential microbial enzymes [58]. Likewise, plant extracts rich in flavonoids and phenolic compounds exert antimicrobial effects through metal ion chelation and disruption of cellular transport processes [59]. Probiotics incorporated into edible coatings suppress pathogenic microorganisms through competitive exclusion and production of bacteriocins, thereby creating unfavorable conditions for spoilage microorganisms [60].

Natural antioxidants, including vitamin E, polyphenols, and plant-derived extracts, are incorporated to inhibit lipid oxidation and protein degradation, the principal causes of undesirable changes in meat color, flavor, texture, and nutritional quality during storage [61]. These compounds function by scavenging free radicals, chelating pro-oxidant metal ions, and stabilizing lipid membranes [62]. For example, catechins and ascorbic acid delay oxidative rancidity while preserving the desirable red color of meat by inhibiting the conversion of oxymyoglobin to metmyoglobin [63].

Plasticizers and emulsifiers are equally important in optimizing film properties. Plasticizers such as glycerol and sorbitol improve flexibility while reducing brittleness by decreasing intermolecular interactions within the polymer matrix [64]. Emulsifiers, including lecithin and Tween 80, promote homogeneous dispersion of hydrophobic compounds, particularly essential oils, thereby improving film uniformity and stability [65, 66].

The concentration of functional additives must be carefully optimized to balance preservation efficacy with sensory acceptability. Essential oils are typically incorporated at concentrations ranging from 0.5% to 5%, depending on their antimicrobial potency and intended application [67]. Similarly, probiotics are generally incorporated at concentrations ranging from 10^6^ to 10^9^ CFU/cm², depending on the microbial strain and desired functionality [68]. Excessive concentrations of essential oils or other highly active compounds may adversely affect the flavor and aroma of meat products. These undesirable sensory effects can be minimized through nanoencap-sulation, which provides controlled release of bioactive compounds while maintaining their antimicrobial and antioxidant activities throughout storage [69, 70].

## FUNCTIONAL PROPERTIES OF EDIBLE COATINGS AND FILMS

**Figure 3:**
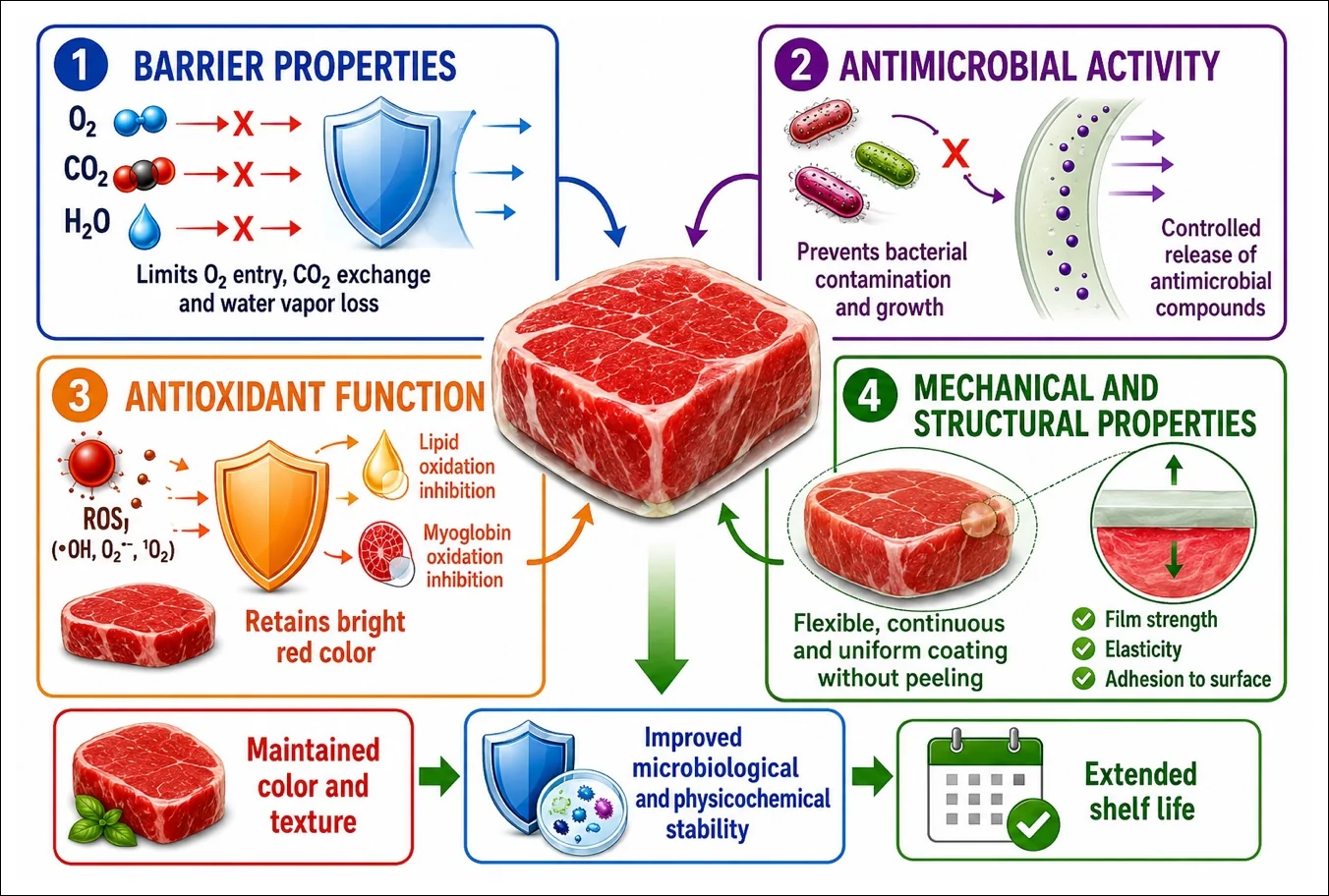
Functional properties and preservation mechanisms of edible coatings and edible films applied to meat products. [Source: Figure prepared by Bima Putra Pratama with the assistance of ChatGPT (OpenAI, GPT-5.5) for AI-assisted figure generation and Canva for graphic design and final layout].

Edible coatings and edible films function not only as physical protective layers but also as active preservation systems that improve the safety, stability, and quality of meat products. Their functional performance is primarily determined by their barrier properties, antimicrobial and antioxidant activities, and mechanical and structural characteristics, all of which contribute to extending shelf-life while maintaining the physicochemical, micro-biological, and sensory quality of fresh and processed meat products. Figure 3summarizes the principal technological functions of edible coatings and films, including inhibition of lipid oxidation, reduction of microbial contamination, regulation of moisture and gas transfer, preservation of color and texture, maintenance of sensory quality, and extension of shelf-life through improved physicochemical and microbiological stability.

### Barrier properties

Barrier properties are among the most important functional characteristics of edible coatings and edible films because they regulate the transfer of gases and water vapor between the meat surface and the surrounding environment. Effective control of O₂ and carbon dioxide (CO₂) permeability is essential for minimizing lipid oxidation, maintaining myoglobin stability, and limiting microbial proliferation during storage [71]. Films with low O_2_ permeability reduce free radical formation and inhibit the oxidation of oxymyoglobin to metmyoglobin, thereby preserving the desirable bright red color of fresh meat [72]. Likewise, controlled CO₂ permeability helps maintain conditions that suppress spoilage microorganisms while supporting a balanced microbial ecosystem on the meat surface [73].

Resistance to water vapor transmission is equally important because it minimizes moisture loss and preserves the texture, tenderness, and juiciness of meat products [74]. Lipid-based coatings, together with protein-lipid and polysaccharide-lipid composite systems, exhibit excellent resistance to water vapor migration, making them particularly suitable for refrigerated storage [75]. In contrast, hydrophilic biopolymers such as proteins and polysaccharides provide excellent O_2_ barrier properties but generally require hydrophobic modifications to improve resistance to moisture transfer [76]. The barrier performance of edible films is further influenced by polymer density, crystallinity, pore structure, and intermolecular interactions. Modern composite and nanocomposite formulations enhance these characteristics by increasing diffusion tortuosity, thereby creating more complex pathways that reduce the movement of gases and water vapor through the polymer matrix [77, 78].

### Antimicrobial activity

The incorporation of antimicrobial agents into edible coatings and edible films represents an effective strategy for extending the shelf-life of meat products by suppressing pathogenic and spoilage microorganisms, including *L. monocytogenes*, *Staphylococcus aureus*, *Salmonella* spp., and psychrotrophic bacteria [79, 80]. In addition to functioning as physical barriers, these systems act as active delivery platforms that gradually release antimicrobial compounds during storage, thereby providing prolonged microbial inhibition [81].

The antimicrobial mechanisms depend on the chemical characteristics of the incorporated bioactive compounds and their interactions with microbial cells [82]. Phenolic constituents present in essential oils disrupt microbial cell membranes, increase membrane permeability, induce oxidative stress, and ultimately cause cell death [83, 84]. Chitosan possesses intrinsic antimicrobial activity through electrostatic interactions between its positively charged amino groups and negatively charged microbial cell surfaces, resulting in membrane disruption, leakage of intracellular components, and inhibition of protein synthesis [85].

The antimicrobial effectiveness of edible coatings also depends on whether the incorporated compounds exert bacteriostatic or bactericidal effects [86]. Bacteriostatic agents inhibit microbial growth without directly killing microorganisms, whereas bactericidal agents actively destroy microbial cells [87, 88]. Distinguishing between these mechanisms is important for selecting appropriate antimicrobial systems for different meat products and storage conditions [89]. In addition, increasing attention has been directed toward preventing biofilm formation on meat surfaces because microbial biofilms substantially increase resistance to conventional antimicrobial treatments [90, 91]. Controlled release technologies, particularly nanoencapsulation, further enhance antimicrobial performance by maintaining sustained release of bioactive compounds throughout storage, thereby ensuring prolonged protection against microbial contamination [92].

### Antioxidant function

Lipid oxidation and myoglobin oxidation are the principal biochemical processes responsible for deterioration of meat quality during storage, particularly under aerobic conditions [93]. Lipid oxidation produces aldehydes, ketones, and other secondary oxidation products that contribute to rancidity, off-flavors, nutritional losses, and reduced consumer acceptability [94]. Simultaneously, oxidation of myoglobin converts oxymyoglobin into metmyoglobin, resulting in undesirable discoloration from bright red to brown and reducing the visual appeal of fresh meat [95]. Edible coatings and edible films enriched with natural antioxidants effectively delay both oxidative processes, thereby extending shelf-life and preserving product quality [96].

Natural antioxidants, including plant polyphenols, essential oils, and vitamin E, protect meat through multiple mechanisms, including scavenging free radicals, neutralizing reactive O_2_ species (ROS), and chelating transition metal ions such as Fe²⁺ and Cu²⁺ that catalyze lipid oxidation reactions [97, 98]. Certain antioxidants exhibit greater affinity for lipid-rich environments, providing enhanced protection against intramuscular lipid oxidation and cellular membrane degradation [99, 100].

Synthetic antioxidants, including butylated hydroxytoluene (BHT) and butylated hydroxyanisole (BHA), have traditionally been used to control oxidative deterioration in meat products; however, increasing consumer preference for naturally derived ingredients has encouraged the development of edible coatings containing plant-derived antioxidants [101]. Natural compounds, such as rosemary extract and catechins, have demonstrated comparable effectiveness in preserving meat color, delaying rancidity, and maintaining overall product quality while addressing consumer concerns regarding synthetic food additives [102, 103].

### Mechanical and structural properties

The mechanical and structural characteristics of edible coatings and edible films are fundamental determinants of their protective performance during meat processing, handling, transportation, and storage [104, 105]. Mechanical properties commonly evaluated include tensile strength, elongation at break, and film thickness, which collectively determine the ability of the film to withstand external stress while maintaining flexibility and providing a continuous protective barrier over the meat surface [106, 107]. Protein-based films, particularly those prepared from gelatin and whey protein, generally exhibit superior tensile strength because of extensive hydrogen bonding and strong intermolecular interactions within the polymer network [108, 109].

Film elasticity is particularly important for coating meat products with irregular or deformable surfaces because it enables the coating to stretch without cracking during handling and storage [110, 111]. Plasticizers such as glycerol and sorbitol improve flexibility by reducing intermolecular forces within the polymer matrix, thereby decreasing brittleness and increasing elongation capacity [112, 113]. Composite formulations combining hydrophilic and hydrophobic biopolymers further optimize the balance between tensile strength and elasticity, producing films that exhibit both structural integrity and sufficient flexibility for practical application [114].

Film thickness is another critical factor influencing barrier performance and overall preservation efficacy [115]. Although thicker films generally provide superior resistance to O_2_ and water vapor transmission, excessive thickness may adversely affect the appearance, texture, and overall sensory acceptability of meat products [116]. Therefore, optimization of film thickness is essential to achieve an appropriate balance between effective barrier protection and desirable sensory characteristics, thereby maximizing the commercial applicability of edible coatings and edible films for meat preservation [117].

## APPLICATIONS IN MEAT AND MEAT PRODUCTS

Edible coatings and edible films have emerged as innovative preservation technologies for fresh and processed meat products because of their ability to extend shelf-life, preserve sensory attributes, and improve microbiological safety [118]. These systems have been successfully applied to a wide range of meat products, including beef, poultry, goat meat, pork, sausages, nuggets, and smoked meats, while also serving as functional components of active packaging systems that provide multiple preservation benefits [119].

### Applications in fresh meat

The application of edible coatings and edible films to fresh meat, including beef, poultry, goat meat, and pork, has gained considerable attention as a sustainable and environmentally friendly preservation strategy [120]. These coatings form a thin protective barrier on the meat surface that slows O_2_ diffusion, minimizes moisture loss, and inhibits the growth of spoilage microorganisms [121]. Consequently, edible coatings help maintain the physicochemical, microbiological, nutritional, and sensory quality of meat during refrigerated storage while reducing dependence on synthetic preservatives [122].

Table 2 summarizes the application of edible coatings and edible films to different fresh meat products, including the coating materials, incorporated bioactive compounds, and their effects on chemical stability, microbiological quality, sensory attributes, and shelf-life [123–133].

**Table 2:** Applications of edible coatings and films for preserving the quality and extending the shelf-life of fresh meat.

**Meat type**	**Edible coating/film material**	**Bioactive compound(s)/Additive(s)**	**Effects on meat quality and shelf-life**	**References**
Beef	Protein-based coatings and films (gelatin and whey protein) and chitosan	Plant-derived antioxidants (rosemary extract, green tea extract, and oregano essential oil)	Inhibits lipid oxidation and myoglobin degradation, maintains the desirable bright red color, reduces TBARS values, and suppresses the growth of *Pseudomonas* spp.	[123–125]
Poultry	Alginate-based edible coatings containing essential oil nanoemulsions	Thyme essential oil and basil essential oil	Delays lipid oxidation, inhibits *Campylobacter* spp. and *Salmonella* spp., and enhances the distribution and penetration of bioactive compounds on the meat surface	[126–128]
Lamb/Goat	Chitosan- and pectin-based edible coatings	Polyphenols (quercetin)	Reduces moisture loss, delays lipid oxidation, preserves color and sensory quality, and minimizes the development of characteristic goaty odor	[129–131]
Pork	Polysaccharide- and protein-based edible coatings and films	Natural antioxidants (tocopherols and fruit peel extracts), bacteriocins, and probiotics	Delays lipid oxidation and off-flavor development, suppresses the growth of *Listeria monocytogenes*, and extends refrigerated shelf-life	[132, 133]

TBARS = Thiobarbituric acid reactive substances. Scientific names are italicized according to taxonomic conventions.

In beef, protein-based films prepared from gelatin or whey protein and enriched with natural antioxidants such as rosemary extract, green tea extract, or oregano essential oil have consistently demonstrated excellent antioxidant activity by suppressing lipid oxidation and delaying myoglobin degradation [123]. These formulations effectively preserve the characteristic bright red color of fresh beef while significantly reducing TBARS during refrigerated storage [124]. Chitosan-based coatings have also demonstrated strong antimicrobial activity against *Pseudomonas* spp., one of the principal spoilage microorganisms responsible for quality deterioration in fresh beef [125].

Poultry meat is particularly susceptible to oxidative deterioration because of its high content of unsaturated fatty acids and elevated metabolic activity [126]. Polysaccharide-based coatings, particularly alginate combined with thyme or basil essential oils, effectively retard lipid oxidation while reducing populations of *Campylobacter* spp. and *Salmonella* spp., two major foodborne pathogens associated with poultry products [127]. Furthermore, nanoemulsified essential oils improve the stability of edible coatings and enhance the penetration and controlled release of bioactive compounds on the meat surface, thereby increasing antimicrobial and antioxidant effectiveness [128].

Goat meat possesses unique lipid and protein compositions that influence oxidative stability and the development of characteristic flavor during storage [129]. Chitosan- and pectin-based coatings effectively reduce moisture loss and lipid oxidation, while incorporation of natural polyphenols such as quercetin improves color stability and preserves desirable flavor characteristics [130]. These coatings also reduce the formation of volatile ketone compounds responsible for the characteristic goaty odor that develops during storage [131].

In pork, edible coatings formulated from protein- or polysaccharide-based biopolymers and enriched with natural antioxidants such as tocopherols and fruit peel extracts effectively inhibit lipid oxidation, thereby minimizing rancidity and undesirable flavor changes [132]. In addition, chitosan coatings containing bacteriocins or probiotics suppress the growth of *L. monocytogenes*, contributing to prolonged refrigerated shelf-life and improved microbiological safety [133].

### Applications in processed meat products

Edible coatings and edible films have been increasingly incorporated into processed meat products, including sausages, nuggets, bacon, and smoked meats, as effective preservation systems that enhance food safety, maintain product quality, and prolong shelf-life [134]. These edible layers, produced from natural biopolymers such as proteins, polysaccharides, and lipids, provide additional protection against oxidative, microbiological, and physical deterioration while maintaining desirable product characteristics [68].

Table 3 summarizes the application of edible coatings and edible films in processed meat products, including coating materials, incorporated bioactive compounds, and their effects on oxidative stability, textural properties, microbiological quality, and storage stability [135–145].

**Table 3:** Applications of edible coatings and films for preserving the quality and extending the shelf-life of processed meat products.

**Processed meat product**	**Edible coating/film material**	**Bioactive compound(s)/Additive(s)**	**Effects on product quality and shelf-life**	**References**
Sausage	Protein-based coatings and films (gelatin and whey protein) and chitosan	Rosemary extract, tocopherol, probiotics, and bacteriocins	Enhances oxidative stability, reduces fat coalescence and lipid rancidity, preserves color and flavor, and inhibits the growth of *Lactobacillus* spp. and *Listeria monocytogenes*	[135–137]
Nugget	Polysaccharide-based coatings and films (alginate and starch) containing essential oil nanoemulsions	Oregano essential oil and thyme essential oil	Controls oil migration, reduces drip loss, improves texture and moisture retention, inhibits *Staphylococcus aureus* and *Escherichia coli*, and decreases oil uptake during frying, thereby lowering fat content	[138–141]
Smoked meat	Protein-based coatings and films (fish protein) and chitosan	Polyphenols (catechin and quercetin) and protective cultures	Enhances surface antioxidant capacity, inhibits xerotolerant microorganisms and pathogens such as *Listeria* spp., improves moisture retention, and prevents case hardening during storage	[142–145]

TBARS = Thiobarbituric acid reactive substances (where applicable). Scientific names are italicized according to taxonomic conventions.

In sausages, which contain complex protein-fat emulsion systems, edible coatings based on milk proteins or chitosan improve oxidative stability while reducing lipid separation during refrigerated storage [135]. The incorporation of natural antioxidants such as rosemary extract and tocopherols effectively delays lipid oxidation, thereby minimizing discoloration, flavor deterioration, and formation of undesirable volatile compounds [136]. Moreover, coatings containing probiotics or bacteriocins inhibit the growth of *Lactobacillus* spp. and *L. monocytogenes*, providing additional protection for ready-to-eat sausage products that are susceptible to post-processing contamination [137].

In nuggets, edible coatings improve product stability by reducing oil migration and preserving structural integrity during frozen storage [138]. Alginate- and starch-based coatings form stable matrixes that reduce drip loss and minimize structural damage associated with repeated freezing and thawing cycles [139]. Incorporation of nanoemulsified oregano or thyme essential oils further enhances antimicrobial activity against *S. aureus* and *E. coli* without adversely affecting sensory quality during chilled or frozen storage [140]. In addition, edible coatings reduce oil absorption during deep-fat frying, resulting in products with improved nutritional quality and lower fat content [141].

Smoked meat products are particularly susceptible to oxidative deterioration because of their relatively high lipid content and exposure to elevated temperatures during smoking [142]. Fish protein- and chitosan-based films enriched with natural polyphenols such as catechin and quercetin significantly enhance antioxidant activity while suppressing the growth of microorganisms that tolerate low-moisture environments [143]. Edible coatings can also function as carriers of protective microbial cultures that produce natural antimicrobial compounds, thereby providing additional protection against *Listeria* spp., one of the most important pathogens associated with ready-to-eat smoked meat products [144]. Furthermore, hydrophilic coatings help retain internal moisture and reduce case hardening in certain smoked and cured meat products [145].

### Integration into active packaging systems

The integration of edible coatings and edible films into active packaging systems represents a significant advancement in meat preservation technology by enhancing microbiological stability, oxidative resistance, and sensory quality throughout storage [17]. Unlike conventional passive packaging, active edible packaging interacts dynamically with the packaged product through controlled release of antimicrobial agents, antioxidants, and compounds that regulate moisture and gas exchange, thereby actively preserving meat quality [146].

Active edible packaging systems are commonly produced from polysaccharides such as chitosan, alginate, and pectin, as well as proteins including gelatin, whey protein, and soy protein. These biopolymers serve as matrixes for controlled release of bioactive compounds, providing prolonged protection against lipid oxidation and microbial contamination [147]. Incorporation of essential oils such as oregano, thyme, and rosemary, nanoemulsified phenolic compounds, and bacteriocins such as nisin has demonstrated selective antimicrobial activity against *L. monocytogenes*, *Salmonella* spp., and *Pseudomonas* spp. while preserving the sensory quality of meat products [148].

This technology also enables the development of multilayer packaging systems consisting of an inner edible layer containing functional bioactive compounds and an outer layer that provides additional mechanical protection [149]. Furthermore, intelligent edible packaging incorporating pH-sensitive indicators or anthocyanin-based colorimetric sensors enables real-time monitoring of meat freshness and early detection of spoilage, providing valuable information for both consumers and the meat industry [150].

Despite these technological advances, widespread industrial implementation remains constrained by several challenges, including maintaining bioactive compound stability during thermal processing, achieving compatibility with conventional packaging materials, complying with food safety regulations, and improving consumer acceptance of edible bioactive ingredients [111]. Continued advances in nanoencapsulation, composite biopolymer engineering, and moisture- or pH-responsive controlled release systems are expected to address many of these limitations and facilitate the broader commercial application of edible active packaging for meat preservation [151].

## EVALUATION METHODS FOR COATING EFFECTIVENESS

Evaluation of the effectiveness of edible coatings and edible films is essential for determining their ability to preserve meat quality, extend shelf-life, and improve food safety. Comprehensive assessment includes chemical, microbiological, sensory, and shelf-life evaluations, which collectively provide a reliable measure of coating performance under different storage conditions [152]. These complementary analytical approaches enable assessment of the capacity of edible coatings to reduce lipid oxidation, suppress microbial growth, preserve physicochemical characteristics, and maintain consumer acceptability throughout storage [28].

Table 4 summarizes the principal analytical methods used to evaluate the effectiveness of edible coatings and edible films applied to meat products, including chemical, microbiological, sensory, and shelf-life assessment parameters [22, 24, 152–163].

### Chemical evaluation

Chemical analyses are widely employed to assess changes in the physicochemical integrity of meat during storage [153]. Measurement of TBARS is one of the most commonly used indicators of lipid oxidation because oxidative degradation of unsaturated fatty acids produces aldehydes, ketones, and other secondary oxidation products responsible for rancidity and quality deterioration [154]. Monitoring pH provides valuable information regarding microbial activity, protein stability, and postmortem biochemical changes, with substantial pH alterations often indicating microbial proliferation or deterioration of meat quality [155].

WHC is another important quality parameter because it reflects the ability of meat to retain its intrinsic moisture during storage and processing, directly influencing texture, juiciness, yield, and consumer acceptability [156]. Effective edible coatings are expected to maintain WHC by minimizing drip loss, reducing moisture migration, and preserving the structural integrity of muscle proteins throughout storage [22].

### Microbiological evaluation

Microbiological assessment is fundamental for determining the effectiveness of edible coatings in controlling spoilage microorganisms and foodborne pathogens [10]. TPC is routinely used to estimate the overall aerobic microbial population on the meat surface, whereas enumeration of specific microorganisms, including lactic acid bacteria and pathogenic bacteria such as *L. monocytogenes*, *Salmonella* spp., and *E. coli*, provides a more comprehensive assessment of microbiological quality and food safety [157].

Edible coatings incorporating antimicrobial compounds, including chitosan, essential oils, bacteriocins, and other natural bioactive agents, are generally expected to significantly reduce microbial populations compared with uncoated control samples [158]. Sustained antimicrobial activity achieved through controlled release systems further enhances microbiological stability during prolonged storage.

### Sensory evaluation

Sensory evaluation is indispensable for determining consumer acceptance of edible coating technologies because preservation strategies must maintain desirable organoleptic properties while extending shelf-life [159]. Sensory assessment is commonly performed by trained panels and consumer groups using standardized evaluation protocols that assess color, aroma, texture, flavor, and overall acceptability.

Maintenance of the characteristic bright red color indicates successful preservation of myoglobin stability, whereas retention of desirable aroma and flavor reflects effective inhibition of lipid oxidation and microbial spoilage [160]. Texture evaluation is equally important because edible coatings should not adversely affect tenderness, surface appearance, adhesiveness, or mouthfeel [161]. An ideal edible coating preserves sensory quality while remaining imperceptible to consumers.

**Table 4:** Methods used to evaluate the effectiveness of edible coatings and films for meat preservation

**Evaluation category**	**Parameter/Method**	**Purpose**	**Indicators assessed**	**References**
Chemical	TBARS	Evaluate the extent of lipid oxidation	Quantifies secondary lipid oxidation products (primarily aldehydes), indicates oxidative rancidity, and assesses oxidative stability, particularly in lipid-rich meat products	[152–154]
	pH	Assess biochemical stability and microbial activity	Reflects postmortem biochemical changes and microbial metabolism; deviations indicate spoilage progression and reduced product stability	[152, 155]
	WHC	Evaluate the ability of meat to retain water	Determines moisture retention, texture, juiciness, cooking yield, and drip loss during storage and processing	[22, 152, 156]
Microbiological	TPC	Assess the total aerobic microbial population	Provides an overall estimate of microbial load and serves as an early indicator of microbial spoilage	[152, 157]
	Specific microbial analysis	Detect spoilage and foodborne pathogenic microorganisms	Evaluates antimicrobial efficacy against target microorganisms, including *Lactobacillus* spp., *Listeria monocytogenes*, *Salmonella* spp., and *Escherichia coli*	[152, 157, 158]
Sensory	Trained sensory panel and consumer evaluation	Assess organoleptic quality and consumer acceptability	Evaluates appearance, color, aroma, texture, flavor, and overall acceptability; detects off-flavor development and color deterioration during storage	[152, 159–161]
Shelf-life assessment	Periodic quality evaluation during storage	Determine the effectiveness of edible coatings and films under practical storage conditions	Monitors changes in chemical, microbiological, physical, and sensory quality at predetermined storage intervals to estimate shelf-life under refrigeration, freezing, or MAP	[24, 152, 162, 163]

MAP = Modified atmosphere packaging; TBARS = Thiobarbituric acid reactive substances; TPC = Total plate count; WHC = Water-holding capacity. Scientific names are italicized according to taxonomic conventions.

### Shelf-life evaluation

Shelf-life studies provide comprehensive information regarding the practical effectiveness of edible coatings under commercial storage conditions [24]. Coated meat products are periodically evaluated throughout storage using chemical, microbiological, and sensory analyses under refrigerated, frozen, or MAP conditions [162]. Monitoring these quality parameters over time enables accurate determination of the ability of edible coatings to preserve product quality, maintain microbiological safety, and delay spoilage.

The integration of chemical, microbiological, and sensory findings provides a comprehensive assessment of preservation performance and facilitates estimation of the commercial shelf-life of coated meat products under realistic storage conditions [163]. These evaluations also provide valuable information for optimizing coating formulations and supporting their industrial implementation in modern meat preservation systems.

## TECHNOLOGICAL CHALLENGES AND LIMITATIONS

Despite the considerable potential of edible coatings and edible films to extend shelf-life while preserving the quality and safety of meat products, several technological, regulatory, economic, and consumer-related challenges continue to limit their widespread commercial application [9]. Addressing these limitations is essential for successful industrial implementation and long-term adoption of this preservation technology.

### Stability of coating materials and bioactive compounds

One of the major challenges is maintaining the chemical and physical stability of both the polymer matrix and incorporated bioactive compounds throughout production, storage, and application [120]. Antimicrobial and antioxidant agents may gradually lose their activity because of exposure to heat, light, O_2_, or interactions with meat constituents such as proteins and lipids [164]. Consequently, reductions in bioactive stability may compromise the long-term effectiveness of edible coatings during storage.

Protein- and polysaccharide-based coatings are also highly sensitive to environmental moisture. Excessive moisture absorption may alter viscosity, induce swelling or cracking, reduce mechanical strength, and impair barrier performance, ultimately decreasing their protective efficacy [165]. Therefore, improving the structural stability of hydrophilic biopolymers remains a major research priority for commercial applications.

### Safety and biodegradability considerations

Edible coatings and edible films must provide effective preservation while remaining safe for human consumption and environmentally sustainable. Although their biodegradability represents a significant environ-mental advantage over conventional petroleum-based packaging materials, rapid biodegradation may compromise coating integrity before the intended shelf-life of the product has been achieved [166]. Consequently, coating formulations must achieve an appropriate balance between biodegradability and functional durability.

Safety evaluation is particularly important when edible coatings incorporate functional ingredients such as nanoparticles, essential oils, or concentrated phenolic extracts. These compounds require comprehensive assessment of potential toxicity, migration into food, and interactions with meat components to ensure compliance with food safety regulations while avoiding undesirable effects on product quality or consumer health [167, 168].

### Industrial scalability and economic feasibility

Although numerous laboratory-scale studies have demonstrated the effectiveness of edible coatings and edible films, translating these technologies to industrial production remains challenging. Large-scale manufac-turing requires optimization of formulation, mixing, casting, drying, coating application, and packaging processes to ensure consistent product quality while maintaining production efficiency [169].

Economic feasibility is another important consideration. High-quality biopolymers, natural bioactive compounds, and advanced technologies such as nanoencapsulation, nanoemulsification, and controlled release systems can substantially increase manufacturing costs, potentially limiting commercial competitiveness [170]. Furthermore, implementation within existing meat processing facilities often requires modification of production lines, specialized coating equipment, and process validation, thereby increasing initial capital investment and operational complexity [119].

### Consumer acceptance and regulatory compliance

Successful commercialization depends not only on technological performance but also on consumer acceptance and market willingness to pay for products containing edible coatings [171]. Consumer perception may be influenced by concerns regarding edible packaging materials, natural bioactive compounds, and particularly nanotechnology-based ingredients. Transparent labeling, effective risk communication, and consumer education will therefore be important for improving market acceptance.

Regulatory approval remains another major challenge. Commercial application requires compliance with national and international food safety regulations governing food additives, edible packaging materials, labeling requirements, migration limits, and product quality standards. Regulatory agencies such as the U.S. FDA, the EFSA, and the Indonesian Food and Drug Authority (BPOM) require comprehensive evidence demonstrating the safety of edible coating components, their interactions with food matrixes, migration behavior, and potential effects on sensory quality before commercial approval can be granted [172–174]. Failure to satisfy these regulatory requirements may significantly delay or prevent market adoption.

### Environmental sustainability

Environmental sustainability is an increasingly important consideration when evaluating edible coating technologies. Although biopolymer-based coatings have considerable potential to reduce dependence on petroleum-based plastics, comprehensive life cycle assessment (LCA) studies are required to objectively compare their environmental performance with conventional packaging systems. Such assessments should evaluate carbon footprint, energy consumption, resource utilization, waste generation, biodegradability, and overall environmental sustainability throughout the product life cycle [175]. These evaluations will provide critical information regarding the true environmental benefits and commercial sustainability of edible coatings and edible films for future meat packaging applications.

## FUTURE PROSPECTS

Edible coatings and edible films have demonstrated considerable potential as sustainable preservation technologies for meat products. Nevertheless, continued technological innovation is required to further improve their functionality, commercial feasibility, and industrial applicability. Current research is primarily focused on three emerging areas: nanoencapsulation, smart edible films, and integration with complementary preservation technologies [9, 11].

### Nanoencapsulation technologies

Nanoencapsulation has emerged as one of the most promising strategies for improving the stability, bioavailability, and controlled release of bioactive compounds incorporated into edible coatings and edible films [175]. This technology involves encapsulating antimicrobial and antioxidant compounds, including essential oils, polyphenols, vitamins, and other functional ingredients, within nanoscale delivery systems that are compatible with the polymer matrix [14]. The exploration of new natural bioactive compounds is also expanding to marine and plant-derived resources. For example, extracts of the sea cucumber *Holothuria edulis *have demonstrated strong antioxidant activity, indicating the broader potential of marine-derived compounds as functional ingredients for future food preservation systems [176]. Similarly, capsaicin has been reported to exhibit antioxidant and antimicrobial activities, although its incorporation into edible coating systems requires further evaluation regarding effective concentration, sensory compatibility, and safety [177].

Application of nanoencapsulation in fresh and processed meat products enables sustained release of bioactive compounds throughout storage, thereby prolonging antimicrobial and antioxidant activity while minimizing undesirable sensory effects associated with high concentrations of free bioactive compounds [178]. In addition to improving controlled release, nanoparticles increase the compactness of the polymer network, enhancing O_2_ and water vapor barrier properties through increased diffusion tortuosity. Consequently, nanoencapsulated edible coatings provide improved protection against lipid oxidation, moisture loss, and microbial deterioration [15].

### Smart edible films

Smart edible films represent an emerging generation of intelligent packaging capable of simultaneously preserving meat quality and monitoring product freshness [179]. These systems incorporate responsive compounds that undergo visible changes, such as color variation, in response to alterations in pH, accumulation of microbial metabolites, or formation of oxidation products during storage [180].

By providing real-time information regarding product quality and safety, smart edible films improve supply chain monitoring, facilitate quality control, and reduce the risk of consumers purchasing or consuming deteriorated meat products [181]. Furthermore, integration of freshness indicators into edible films enables simultaneous preservation and quality monitoring without compromising barrier performance or the activity of incorporated antimicrobial and antioxidant compounds [182]. In the meat industry, the increasing use of Internet of Things-based sensors, rapid detection methods, and blockchain-supported traceability within food safety management systems further illustrates the potential for integrating smart films with digital monitoring and HACCP-based control strategies [183].

### Integration with emerging preservation technologies

Combining edible coatings and edible films with other advanced preservation technologies offers significant opportunities to achieve synergistic preservation effects through multiple complementary mechanisms [184]. Integration with technologies such as HPP, MAP, ultraviolet-C (UV-C) irradiation, and cold plasma treatment has demonstrated considerable potential for improving microbiological safety while maintaining desirable physicochemical and sensory characteristics.

For example, edible coatings containing antimicrobial compounds combined with HPP or cold plasma treatment can substantially reduce microbial populations without adversely affecting meat quality [185]. Similarly, combining edible films with MAP enables more effective regulation of O_2_ and CO_2_ concentrations, thereby reducing microbial growth, delaying lipid oxidation, and extending product shelf-life while maintaining food safety [186]. Such hurdle-based preservation strategies provide more robust protection than individual preservation methods alone and are expected to become increasingly important for both fresh and processed meat products [187].

### Technology readiness and future research directions

Although these emerging technologies have demonstrated promising laboratory-scale performance, additional research is required before widespread industrial implementation can be achieved. Nanoencapsulation systems, smart edible films, and integrated preservation technologies are currently considered to be at intermediate stages of technological development, generally corresponding to Technology Readiness Levels (TRLs) 4-6, indicating that further pilot-scale validation and industrial demonstration are required before commercial deployment [187].

Future research should therefore focus on improving manufacturing scalability, reducing production costs, optimizing long-term stability, and validating performance under commercial processing and distribution conditions. Comprehensive economic analyses, standardized industrial production protocols, and large-scale field studies will be essential to demonstrate commercial feasibility.

Equally important are harmonized regulatory frameworks governing the use of edible coating materials, bioactive compounds, and nanotechnology in food packaging. Greater international alignment among regulatory authorities, including the U.S. FDA and the EFSA, would facilitate product approval, international trade, and broader commercial adoption. In parallel, consumer education and transparent communication regarding the safety and environmental benefits of edible coatings will be critical for improving public acceptance. Addressing these scientific, technological, regulatory, and socioeconomic challenges will accelerate the transition of edible coatings and edible films from promising research innovations to commercially viable, sustainable preservation technologies for the global meat industry.

## CONCLUSION

ECs and EFs have emerged as promising, sustainable preservation technologies that effectively improve the quality, safety, and shelf-life of fresh and processed meat products. The evidence synthesized in this review demonstrates that protein-, polysaccharide-, and lipid-based biopolymers provide complementary barrier functions against O_2_ and water vapor while serving as efficient carriers for antimicrobial and antioxidant compounds. Composite and nanocomposite formulations further enhance mechanical strength, barrier performance, and controlled release of bioactive agents, thereby reducing lipid oxidation, suppressing microbial proliferation, preserving desirable color and texture, and maintaining the overall sensory quality of meat during storage. Recent advances in nanoencapsulation, smart EFs, and the integration of ECs and EFs with MAP, HPP, UV-C treatment, and cold plasma have further expanded their functionality and commercial potential.

From a practical perspective, ECs and EFs offer the meat industry environmentally sustainable alternatives to synthetic preservatives and petroleum-based packaging materials. Their application can extend product shelf-life, reduce postharvest losses, improve food safety, preserve nutritional and sensory quality, and support increasing consumer demand for clean-label and environmentally responsible meat products. Furthermore, their integration into active and intelligent packaging systems provides opportunities for real-time quality monitoring, improved inventory management, and more efficient meat distribution throughout the supply chain.

A major strength of this review is its comprehensive synthesis of recent advances in EC and EF technologies, integrating current knowledge on biopolymer materials, composite and nanocomposite formulations, functional additives, preservation mechanisms, practical applications, evaluation methods, technological challenges, regulatory considerations, and emerging innovations. By critically consolidating evidence from recent studies, this review provides an updated scientific framework that may assist researchers, food scientists, and the meat industry in developing and implementing effective preservation strategies.

Despite these advances, several limitations remain. Most available studies have been conducted under laboratory or pilot-scale conditions, with relatively limited validation under commercial processing, packaging, transportation, and retail environments. Considerable variability in coating formulations, evaluation metho-dologies, storage conditions, and meat types also limits direct comparison among studies. In addition, challenges associated with industrial scalability, production costs, long-term stability of bioactive compounds, regulatory approval, nanoparticle safety, and consumer acceptance continue to constrain large-scale commercialization.

Future research should prioritize the development of standardized EC and EF formulations and harmonized evaluation protocols, together with large-scale industrial validation studies under commercial production conditions. Further investigations should focus on improving manufacturing scalability, reducing production costs, optimizing controlled release systems, and conducting comprehensive safety assessments of nanomaterials. Additional studies addressing LCA, regulatory harmonization among authorities such as the FDA and EFSA, and consumer acceptance will be essential to facilitate commercialization. Integration of ECs and EFs with smart packaging technologies, intelligent sensing systems, and emerging nonthermal preservation technologies is expected to further improve preservation efficiency and accelerate industrial adoption.

In conclusion, ECs and EFs represent scientifically sound and environmentally sustainable preservation technologies with substantial potential to transform modern meat preservation. Continued interdisciplinary research integrating food science, material engineering, nanotechnology, and packaging innovation will facilitate the transition of these technologies from laboratory research to commercial practice. Successfully addressing the remaining technological, economic, regulatory, and consumer-related challenges will enable ECs and EFs to become integral components of next-generation sustainable meat preservation systems, contributing to improved food safety, reduced environmental impact, and enhanced resilience of the global meat industry.

## DATA AVAILABILITY

No new data were generated or analyzed in this review article. All information supporting the findings of this article is derived from the published literature cited in the manuscript.

## GENERATIVE AI DECLARATION

The authors used generative artificial intelligence (ChatGPT, OpenAI, GPT-5.5) to assist with language editing and the preparation of selected figures, while Canva was used for graphic design and figure layout. All AI-assisted content was critically reviewed, revised, and verified by the authors. The literature search, selection of relevant studies, synthesis of evidence, interpretation of findings, and formulation of conclusions were conducted by the authors, who assume full responsibility for the accuracy, originality, and integrity of the article. No artificial intelligence tool was credited as an author.

## AUTHORS’ CONTRIBUTIONS

BPP, ARK, MHE, and WT: Conceptualization, literature review, manuscript drafting, and original draft preparation. SSP, DAAK, and IFM: Manuscript review, editing, and critical revision for important intellectual content. BB, DAP, and SR: Literature acquisition, manuscript preparation, and critical review. ENU, IBM, BWKW, and EF: Figure preparation, reference management, visualization, and manuscript editing. All authors read and approved the final version of the manuscript.
